# Detecting positive selection from genome scans of linkage disequilibrium

**DOI:** 10.1186/1471-2164-11-8

**Published:** 2010-01-05

**Authors:** Chad D Huff, Henry C Harpending, Alan R Rogers

**Affiliations:** 1 Department of Human Genetics, Eccles Institute of Human Genetics, University of Utah, Salt Lake City, UT, USA; 2 Department of Anthropology, University of Utah, Salt Lake City, UT, USA

## Abstract

**Background:**

Though a variety of linkage disequilibrium tests have recently been introduced to measure the signal of recent positive selection, the statistical properties of the various methods have not been directly compared. While most applications of these tests have suggested that positive selection has played an important role in recent human history, the results of these tests have varied dramatically.

**Results:**

Here, we evaluate the performance of three statistics designed to detect incomplete selective sweeps, LRH and iHS, and ALnLH. To analyze the properties of these tests, we introduce a new computational method that can model complex population histories with migration and changing population sizes to simulate gene trees influenced by recent positive selection. We demonstrate that iHS performs substantially better than the other two statistics, with power of up to 0.74 at the 0.01 level for the variation best suited for full genome scans and a power of over 0.8 at the 0.01 level for the variation best suited for candidate gene tests. The performance of the iHS statistic was robust to complex demographic histories and variable recombination rates. Genome scans involving the other two statistics suffer from low power and high false positive rates, with false discovery rates of up to 0.96 for ALnLH. The difference in performance between iHS and ALnLH, did not result from the properties of the statistics, but instead from the different methods for mitigating the multiple comparison problem inherent in full genome scans.

**Conclusions:**

We introduce a new method for simulating genealogies influenced by positive selection with complex demographic scenarios. In a power analysis based on this method, iHS outperformed LRH and ALnLH in detecting incomplete selective sweeps. We also show that the single-site iHS statistic is more powerful in a candidate gene test than the multi-site statistic, but that the multi-site statistic maintains a low false discovery rate with only a minor loss of power when applied to a scan of the entire genome. Our results highlight the need for careful consideration of multiple comparison problems when evaluating and interpreting the results of full genome scans for positive selection.

## Background

Until a few years ago, studies of positive selection have been limited to sequence data from a single gene covering only a few thousand nucleotides. Now that detailed genetic maps of human variability are available in many populations, it is possible to measure the signature of positive selection on a genomic scale [1,2]. Traditional tools for detecting selection are not applicable to these large SNP datasets, as most traditional tests require sequence data with no ascertainment bias. However, with dense SNP coverage across the genome, it is now possible to accurately measure the decay of linkage disequilibrium (LD) over long genomic distances, opening the door for new tests that can detect the fingerprint of selection across hundreds of thousands of nucleotide positions. Most of the tests that measure this signal of selection have been constructed using one of two basic statistics, Extended Haplotype Homozogosity (EHH) and Fraction of Recombinant Chromosomes (FRC) [3,4]. Variants of both statistics have been used in multiple whole genome scans to provide a global view of recent positive selection in humans.

Most of the discussion surrounding these genome scans has focused on the similarities of their results, since all indicate that positive selection has been a surprisingly important force in recent human evolution [4-7]. However, beneath the broad picture lie curious differences in the results of the two approaches. In 2006, Wang et al. identified 1799 candidate genes as potential targets of recent positive selection in a scan of the human genome based on the FRC statistic. Later that year, Voight et al. [5] identified 431 candidate genes in a similar genome scan using the iHS statistic, which is based on EHH. Both groups calculated a summary statistic at each site that measured the LD associated with that site, and then aggregated those statistics to identify candidate loci from the outliers of the empirical distribution, with Voight et al. including 1% of the distribution and Wang et al. including 1.6%. So although the Wang et al. study was only slightly less restrictive, it identified over four times the number of candidate loci. One possible explanation is that FRC is a more powerful statistic for detecting recent positive selection. However, Voight et al. estimated that the power of iHS to detect recent positive selection was approximately 33% for the range of allele frequencies considered in Wang et al. If their estimate is accurate, even if the power of the FRC test is 100%, the discrepancy between the two tests cannot fully explained. Additional genome scans have demonstrated that the differences in these results are not artifacts, and instead represent stable differences between the two statistics [6,8].

While several studies have estimated the power of EHH statistics to infer positive selection, the statistical power of FRC has not yet been explored. To address this gap, we use simulated data to compare the properties of FRC and EHH statistics. We first examine the power of the single-site statistics of each method under explicit null models of neutrality and alternative models of selection. We then estimate the false positive rate, power, and false discovery rate of each test when applied to an empirical distribution of its respective statistic based on a combination of neutral and selected loci.

The available computational methods for simulating genealogies cannot easily model complex demographic scenarios combined with the presence of positive selection. Most methods require a single population of constant size. This is problematic when evaluating the statistical power of LD-based tests in the presence of positive selection, as population bottlenecks and subdivision can create LD that mimics that generated by selection. Here, we introduce a new approach for simulating positive selection in complex population histories with subdivision, migration, bottlenecks, and expansions in a coalescent framework. With this approach, we first generate a set of potential allele trajectories for the favored allele using forward-in-time simulations. Then for each backwards-in-time simulation, we select an allele trajectory at random and condition the coalescent simulation on the population sizes and migration history of the favored allele as specified by the allele trajectory (see Methods).

## Results

In our analysis, we considered one test derived from the FRC statistic, ALnLH, and two tests derived from the EHH statistic, LRH and iHS [3-5]. To evaluate the effects of population history on the power of each of these statistics, we considered four demographic models: constant population size, expansion, expansion with migration, and bottleneck with migration. For the final three models, we obtained parameter values from the best fitting model in [9]. From this model, we used Africa to represent the expanding population and Europe to represent the bottlenecked population. For histories with migration, we allowed low levels of migration between Europe, Asia, and Africa as specified in their model [9]. For selection models, we used the estimate for the average strength of recent positive selection in humans of s = 0.022 from [8]. We set the origin generation of the favored allele for each model to produce an average allele frequency of approximately 0.5, which met our goal of providing coverage for allele frequencies between 0.2 and 0.8.

**Figure 1:**
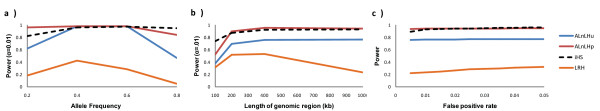
**Power to detect selection from single-site statistics with a constant recombination rate**. For all figures, the power was averaged across 4 population histories of constant size, expansion, expansion with migration, and bottleneck with migration. Both ALnLH_p _and iHS performed quite well in most models. The power of LRH was consistently lower than the other statistics. Neutral simulations for each set of simulation parameters provided the critical values for each statistic. **a.** Power to detect selection for allele frequencies between 0.2 and 0.8 with a simulated region of 1 Mb at a significance level of 0.01. ALnLH_p _and ALnLH_u _were equivalent when allele frequencies were close to 0.5, but the power of ALnLH_u _drops by 40% with allele frequencies of 0.2 and 0.8. **b.** Power to detect selection in simulated regions of 0.1 Mb to 1 Mb. The power was calculated from an equal proportion of allele frequencies 0.2, 0.4, 0.6, and 0.8 for the favored allele at a significance level of 0.01. The average power increased substantially for ALnLH and iHS out to nucleotide lengths of 400 Kb, beyond which there was little improvement. **c.** Power to detect selection for significance levels of 0.005 to 0.05 with simulated region of 1 Mb and an equal proportion of allele frequencies 0.2, 0.4, 0.6, and 0.8 for the favored allele. The average power of ALnLH_p _and iHS was over 0.9 for significance levels of 0.01 or greater.

Throughout the analysis, we calculated two versions of the FRC statistic. As originally presented by Wang et al., FRC is calculated from unphased data using the individuals homozygous for each allele at the focal site [4]. However, the two EHH tests we evaluated, LRH and iHS, are calculated from phased data [3,5]. This introduces a complication when directly comparing the statistics with simulated data, since ALnLH will have lower power than it would otherwise because it ignores information about phase that is available to the other two tests. To account for this, we calculated both a phased and unphased version of the statistic, ALnLH_p _and ALnLH_u_, with the phased statistic using information from both homozygotes and heterozygotes to infer FRC. As shown in Figure 1a, the power of the unphased statistic was much lower than the phased statistic when one of the alleles is relatively rare, but as the allele frequency approaches 0.5 the two statistics were essentially equivalent. Throughout our analysis, we make the simplifying assumption that gametic phase is known, when in practice it can only be estimated. While this assumption may bias our evaluation of ALnLH_u_, the effect should be small given the accuracy of current phase estimation technology and that ALnLH_u _ignores information from all heterozygote comparisons [4,10,11].

**Figure 2:**
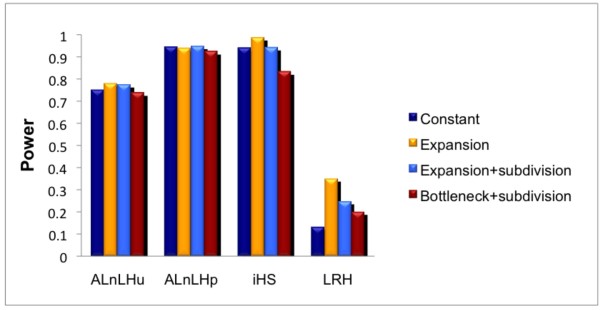
**Power to detect selection from single-site statistics for various demographic models with a constant recombination rate**. All statistics perform well under all 4 population histories. The only statistic notably sensitive to population history was iHS, which performed particularly well in models with expansion and relatively worse in models with bottlenecks and migration. The simulated region was 1 Mb in length with an equal proportion of allele frequencies 0.2, 0.4, 0.6, and 0.8 for the favored allele at a significance level of 0.01.

**Figure 3:**
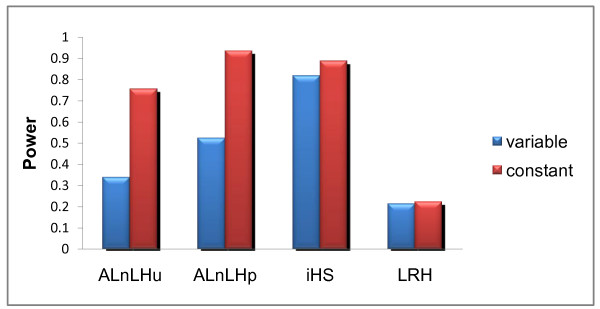
**Effects of variable recombination rate on the power of selection statistics**. The variable recombination rate reduced the power of iHS by 8% and the power of ALnLH_p _by 46%. The locus recombination rate for each simulation was set to an exponential random variate with mean equal to 1 cm/Mb. For other simulation parameters, see Figure 2.

In general, the properties of iHS and ALnLH_p _were similar when the recombination rate was constant (Figure 1). The power of both tests increased substantially with the size of the simulated region out to 400 Kb, beyond which there was little improvement, as shown in Figure 1b. Both statistics performed very well even at low critical levels, with an average power of over 0.9 at the 0.01 level (Figure 1c). The statistics also perform well across the range of allele frequencies we tested, with an average power of over 0.8 at the 0.01 level for allele frequencies between 0.2 and 0.8 (Figure 1a). Both statistics maintained high power across all of our demographic models, though iHS was more sensitive to expansions, bottlenecks, and migration (Figure 2). The performance of the statistics diverged when we introduced variable recombination rates to the models. While both tests were negatively impacted, the effect on ALnLH_p _was much greater, as shown in Figure 3. On average, the power of ALnLH_p _dropped by 46%, while iHS dropped by only 8% for (Figure 3). This directly reflects the strength of the internal controls for local recombination rates within each test. For iHS, there is no measure of global recombination rate, and the measurement of LD is based solely on the relative difference in LD between the two alleles at each site [5]. For ALnLH_p_, the global recombination rate is based on the observed decay of LD at G6PD and the genome deviation from the G6PD model [4]. The test controls for local recombination rate by ignoring all sites where the observed LD is greater than 1 standard deviation above the mean for both alleles. Therefore, positive selection is difficult to detect in regions with high recombination rates, as discussed by Wang et al.[4] in their analysis of positive selection at the DRD4 gene.

For the results presented above, we calculated a statistic for each SNP and evaluated the power to detect selection, with the null hypothesis of neutrality and the alternative hypothesis of strong positive selection acting on the SNP in question. This is an appropriate test for positive selection when the investigator has a prior hypothesis about the potential influence of natural selection and when there are a small number of candidate loci. However, as we demonstrate below, when this simple strategy is applied to an uninformed scan across the genome, it introduces a multiple testing problem that heavily weights the significant results toward false positives. The testing methodology that Voight et al. [5] employed for iHS addresses this problem by binning the genome into 100 Kb segments, and then calculating the fraction of SNPs in each segment with |iHS| greater than 2.0 as their test statistic. This approach takes advantage of the tight linkage of genetic hitchhikers near the favored locus to reduce the number of tests from the number of SNPs in the study to the number of 100 Kb regions in the genome. Their candidate genes were those in regions with the highest fraction of significant iHS scores, taking the top 1% of the empirical distribution. By lowering the criteria for a significant iHS score and considering the total fraction of significant results, they were able to test each 100 Kb region one time at the 0.01 level. In contrast, Wang et al. set a higher threshold for a significant result and tested each SNP individually, taking the top 1.6% of the distribution. All genes within 100 Kb of a significant result were included as candidate genes, which resulted in potentially hundreds of tests at the 0.016 level for each 200 Kb region [4].

**Figure 4:**
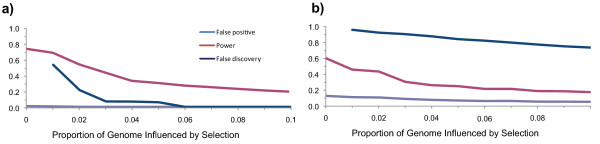
**Power, false positive rates, and false discovery rates for a) iHS and b) ALnLH_u_**. To obtain critical values, we combined loci from neutral simulations with loci from selection simulations in proportion to the fraction of the genome influenced by positive selection [12]. Our implementation of each test followed the methodology employed in the empirical genome scans from [5] (iHS) and Wang et al., 2006 (ALnLH). The false discovery rate was between 0 and 0.53 for iHS, and between 0.74 and 0.96 for ALnLH_u_. To approximate the histories of the populations in the original studies, we combined results from simulations of African and European populations based on the best fitting model in [9]. The locus recombination rate for each simulation was set to an exponential random variate with mean equal to 1 cm/Mb. For other simulation parameters, see Figure 2.

Figure 4 illustrates the different effects of the two approaches. For these results, we follow Teshima et al. [12] in combining data from neutral simulations with selection simulations to evaluate the performance of each empirical test. Since both methods depends heavily on the fraction of the genome that has been affected by positive selection, we allowed this fraction to vary between 0 and 0.1, reporting the corresponding range of values for power, false positive rates, and false discovery rates (Figure 4). Here we distinguish between the false positive rate and the false discovery rate, with the first equal to the rate of false positives for each test, and the second equal to the rate of false positives among all of the statistically significant results. Since both tests were designed to identify candidate genes from a full genome scan, for this analysis we evaluated the statistical properties of the tests at the gene level rather than the SNP level. For the iHS test, we make the simplifying assumption that each gene is contained in a single 100 Kb region. With one 100 Kb test statistic for each gene evaluated at the 0.01 level, the false positive rate per gene is at most 0.01. For ALnLH_u_, we treat the test statistic for each SNP within a 200 Kb region as a separate (but not independent) test for each gene. While the false positive rate per SNP was 0.016 for ALnLH_u _[4], we estimate that the false positive rate per gene was between 0.05 and 0.13. Therefore, of those candidate genes identified by Wang et al., we estimate that a fraction between 0.74 and 0.96 are false positives (Figure 4b). In comparison, we estimate that the false discovery rate for each gene in Voight et al. was between 0 and 0.53. Despite the higher false positive rate, we estimate that the power was approximately 25% lower in the Wang et al. test due to the issues with uncertain phase and variable recombination rates discussed above.

## Discussion

From our evaluation of false discovery rates, we can estimate the number of false discoveries for each genomic scan. Of the 1799 candidate genes identified by Wang et al. [4], we estimate that 1331 to 1727 of those were false discoveries. For Voight et al. [5], we estimate that there were 0 to 231 false discoveries over 431 candidate genes. The estimates for true discoveries are then 72 to 468 for Wang et al. [4], and 200 to 431 for Voight et al. [5]. So after adjusting for false discoveries, the two studies are in close agreement. Given our true discovery and power estimates for iHS, we estimate that there are between 600 and 1000 variants with an allele frequency of at least 0.2 that have been influenced by strong recent positive selection in the HapMap phase 2 populations.

While the single-site statistics used in these studies perform equally well under simulations with constant recombination rates, several factors inhibited the performance of ALnLH. These factors primarily involve implementation details of the test and not the properties of the FRC statistic itself. Since both ALnLH and iHS methods measure the long range LD for each allele at each focal site, it may be possible to design a test based on the FRC statistic that matches or exceeds the performance of iHS using the Voight et al. implementation as a template [5]. Five features that should be included in such a test are local controls for recombination rate, standardization for allele frequency, population specific critical regions, external inference of gametic phase, and the aggregation of results at nearby loci to mitigate multiple testing problems. While a future FRC test may prove more valuable, the false positive and false discovery rates are too high in the current ALnLH implementation to provide a useful set of candidate genes in genomic scans.

Throughout our analysis of EHH statistics, iHS consistently outperformed LRH. Since specific guidelines are not available for determining the core haplotype region and level of EHH decay for LRH, we may have underestimated the power of LRH. However, we tested 4 sets of parameter values using examples in Sabeti et al. as a guide [3], and none of the tests were able to match the performance of iHS in any of our scenarios.

Our estimates for the power of the iHS test were consistently higher than those reported in Voight et al. [5], but it is important to distinguish between our single-site analysis vs. our site-aggregation analysis when comparing the two results. Figures 1, 2, and 3 report the power of the single-site statistic, which is based on one iHS value measuring the decay of LD surrounding one SNP. This is not directly comparable to the power analysis in Voight et al., which was based on the aggregation of 51 iHS scores for SNPs near the favored allele [5]. This aggregation strategy successfully mitigates the multiple testing problem inherit in a full genome scan by incorporating information from potential genetic hitchhikers near the favored allele. However, as demonstrated in the comparisons between Figures 3 and 4, the power of the site-aggregation test is appreciably lower than the single-site test. This tradeoff is worthwhile for uniformed genome scans involving large numbers of SNPs, since it reduces the number of tests by one or more orders of magnitude. However, candidate gene studies that involve only a few potential targets of selection do not suffer from the same multiple testing problems as full genome scans. For these studies, the single-site iHS test is a better choice, providing an average power of 0.81 at the 0.01 level according to our estimates (Figure 3).

There are two other considerations when comparing the power analysis from Voight et al. [5] with the results from this study. First, Voight et al. [5] established critical values from null models with histories of population bottlenecks, but tested those values against selection models where the population size was constant. Because population bottlenecks also introduce LD, this resulted in conservative critical regions and lower power. Second, our strategy for simulating ascertainment bias resulted in higher SNP density and more low frequency alleles compared to Voight et al. [5], which probably elevated the power of the test in our analysis when the favored allele was relatively rare.

As pointed out by Przeworski et al., empirical scans for selection will miss many selection events when they are applied to genomes that have been heavily influenced by recent positive selection [13]. This is evident in Figure 4a, where the power of iHS is 0.74 when selective sweeps are very rare, 0.69 when 1% of the genome is influenced by positive selection, and drops to 0.33 when just 5% of the genome influenced by selection. This effect could be mitigated by choosing critical values from a subset of the genome that has a smaller proportion of recent selection events. We expect *a priori *that nongenic regions are less likely to be targeted by selection. This expectation is supported in Voight et al. [5], where they demonstrated a highly significant enrichment for genic regions within the group of loci identified as potential targets of positive selection (p < 1E-20). By establishing critical regions from nongenic regions, it may be possible to substantially improve the power of genome scans for recent positive selection with only a small increase in false positives.

## Conclusions

In agreement with previous findings, our results demonstrate that the multi-site iHS test is an excellent test for detecting incomplete selective sweeps in full genome scans, with power between 0.33 and 0.74 and false discovery rate between 0 and 0.53 at the 0.01 level. In comparison, the power of the ALnLH test in full genome scans was approximately 25% lower with a false discovery rate between 0.74 and 0.96. However, the statistical properties of the two statistics are quite similar when applied to a single site in a candidate gene test, with power of over 0.8 at the 0.01 level, demonstrating the importance differences in the adjustments made for multiple tests in full genome scans. Our results highlight the need for careful consideration of multiple comparison problems when evaluating and interpreting the results of full genome scans for positive selection. The algorithm we present for simulating genealogies influenced by positive selection will allow for more thorough exploration of complex demographic scenarios when evaluating methods for detecting positive selection.

## Methods

### Simulating the allele trajectory

To simulate positive selection, we employed the coalescent framework first proposed by Kaplan et al. [14], where the selected and neutral alleles are treated as two subdivided populations. In this method, the trajectory of the favored allele is determined separately through model or simulation, which provides the population sizes of the two allelic classes throughout the coalescent simulation. Though there are a variety of existing methods for generating the trajectory of the favored allele, most are limited to simple models of demography and selection. The original method of Kaplan et al. [14] models strong balancing selection by assuming that allele frequencies remain constant. Braverman et al. [15] introduced a model of directional selection, but the trajectory path is deterministic. Stochastic simulations of the trajectory have generally been limited to backward time Moran models, which require a single population of fixed size [13,16-18]. Slatkin proposed an importance-sampling method that weights realizations of a reversed Wright-Fisher model according to the conditional probability of the trajectory path in forward time given the observed genetic data [19]. This model allows for variable population size, and could be extended to include population subdivision and migration. However, the method is computationally intensive and the introduction of n subpopulations with migration would increase computational complexity by a factor of n^2^+n. Pickrell et al. [7] adopt a hybrid approach, where a single population is initialized by coalescent simulation until the first population split. From that point on, the simulation occurs in forward time using Wright-Fisher drift [7]. While this method can model complex demography, it does not allow for conditioning on the desired allele frequency of the favored allele. It also requires the simulation to track each recombinant haplotype in each subpopulation, and as such is computationally intensive even for relatively small genomic regions.

In the interest of developing a more flexible method, we introduce a new importance-sampling method based on forward Wright-Fisher drift. Consider a sample of n sequences from a single subpopulation, x of which carry a favored allele that originated t generations ago with a selection coefficient of s. We would like to draw randomly from the trajectories that produce x modern copies of the favored allele in a sample of size n. To accomplish this, we simulate the forward trajectory of the favored allele, continuing until the allele is lost, becomes fixed, or until t generations have passed. Let p equal the frequency of the allele in the subpopulation in the final generation. Then the importance weight for our desired distribution is the binomial likelihood function:

Because Wright-Fisher drift is a Markov process, the importance weight depends only on the allele frequency in the final generation. In contrast, Slatkin's method employs a backward process that is only a rough approximation to Wright-Fisher drift, so the sampling weight must be calculated over the entire history of the two alleles with a separate term for each population and for each potential migration path in each generation [19].

Because the allele trajectory is generated from Wright-Fisher forward simulation, this method can seamlessly model complex demographic scenarios that include bottlenecks, expansions, and population subdivision with migration. The biggest downside to this flexibility is the potential for choosing parameter values that rarely result in population allele frequencies that are near the observed frequency in the sample. This concern must be evaluated when choosing parameter values, as some will require a prohibitive number of forward simulations to cover the sample space. However, all of the backward time methods are approximations to a forward Wright-Fisher process, and are meant to model natural processes that clearly occur in forward time, so this method is adequate for exploring most relevant models of positive selection and demography. For models where the sample allele frequency is particularly unlikely, Slatkin's method will be preferable since it involves a backward process conditioned on the sample [19].

For the results presented here, we set s and t to fixed values, though in principle they could be set to random variates in each forward iteration, reflecting uncertainty around estimates of selection strength and allele age. If t is a random variable, each origin generation-subpopulation must be weighted by its respective population size to reflect the probability that a new mutation originates in that generation [19].

### Coalescent simulations

We assumed all recombination events were crossovers, where a crossover occurs with the favored or neutral allele with probability proportional to the frequency of the alleles in the subpopulation [20,21]. For models with variable recombination rates, we followed Przeworski et al. [13] in setting the recombination rate to an exponential random variate in each simulation, with mean equal to the rate of recombination in our constant models, 1 cm/Mb. We adopted the implementation details of the coalescent process from [17], storing each generation in a lookup table indexed by the cumulative hazard of coalescence. To account for population subdivision, we introduced a subpopulation dimension to the coalescence table.

The trajectory of the favored allele was generated under a model where the migration rates are constant between subpopulations for each epoch. However, since a trajectory is in part a realization of this random process, we could not assume constant migration rates in a coalescent simulation based on a particular trajectory. The number of individuals of each genotype migrating to and from each population in a given generation is determined by the forward simulation and is therefore treated as a constant during the backward simulation. The individual migrants themselves are, however, chosen at random during the backward simulation. To implement this process, we introduced two migration lookup tables. The first table was analogous to the coalescence lookup table, storing the cumulative hazard of migration out of a given subpopulation for each allele. We used the second table to determine the destination subpopulation of a migrant, by storing the conditional probability of migrating from an origin subpopulation to a destination subpopulation given that a migration event occurred out of the origin subpopulation in a particular generation. Expanding on Coop and Griffith's method, we accessed the coalescence and migration lookup tables with uniform random variates to generate the waiting time until the next event for each subpopulation-allele combination [17]. We then generate the waiting time until the next recombination event from an exponential random variate. Then from the memoryless property of the exponential distribution, the next event to occur is the event with the shortest wait time [17,20].

### Ascertainment bias

To introduce ascertainment bias to the simulated data, we developed a procedure to model the process in the Perlegen dataset. In their SNP discovery process, they identified all polymorphic sites in a fully sequenced subsample, then genotyped those sites in a larger sample [22]. To replicate their procedure on simulated data, we randomly assigned mutation events to the tree under an infinite sites model using a mutation rate of 2.2E-8 per nucleotide per generation [23]. We then designated 13-33% of the chromosomes in each simulation as the ascertainment subsample [22]. We excluded all mutations that were not polymorphic in the subsample. To generate diploid genotypes for calculating FRC, we grouped the simulated chromosomes into randomly chosen pairs.

### Statistics

EHH is defined as the probability that two chromosomes in a sample share the same haplotype for a given set of SNPs [3]. Sabeti et al. [3] introduced the long range haplotype test (LRH), which is calculated by dividing EHH on a core haplotype by EHH among all samples not containing the core haplotype. Since explicit guidelines for identifying the core haplotype were not available, we tested two criteria. Our core haplotype region was then either a fixed 15 Kb region surrounding the focal site, or the first 8 SNPs nearest the focal site with minor allele frequencies greater than 0.05, including the focal site. The core haplotype was then the largest haplotype in the core haplotype region. We based these criteria on the size of the G6PD region and the simulation methodology employed in [3]. We calculated LRH for both methods at the furthest distance where the EHH was greater than either 0.25 or 0.05 at core haplotype. In our tests, the 15 Kb regions with an EHH cutoff of 0.25 had the highest average power of the options we considered, so we only report those results. For both LRH and iHS, we measure EHH from the expected homozygosity given the allele frequencies of each haplotype rather than observed homozygosity.

Voight et al. [5] introduced the integrated EHH (iHH), which is the integral of the observed decay of EHH away from a particular allele, summing over both directions until EHH is less than 0.05. To obtain their single-site statistic, iHS, they divide the value of iHH at the ancestral allele by the value of at the derived allele and then take the natural log. Finally, they standardize iHS by subtracting the expectation and dividing by the standard deviation, which are conditioned on the frequency of the derived allele. This final step accounts for the frequency of the allele, since low frequency derived alleles are younger and as such will be associated with longer LD blocks.

FRC is the fraction of inferred recombinant chromosomes between two sites within a sample [4]. Throughout their analysis, the decay of FRC at G6PD is used as a model for recent positive selection, and each site is measured by how closely it matches the model. Their formula for this model was derived by fitting a sigmoid to the observed decay of FRC at G6PD:

where X is the distance from the focal site.

For a given allele at a focal site, Wang et al. calculate FRC separately for each site within 500 Kb of the focal site with a minor allele frequency greater than 0.1 [4]. They then input each array of FRC statistics into a pseudo-likelihood function to measure the goodness-of-fit to the G6PD model under the assumption that FRC values are normally distributed. This likelihood is adjusted for allele age, as described below.

Positively selected alleles that are much younger than G6PD will, in general, have larger LD blocks surrounding the selected allele. If the likelihood calculation were left unadjusted, this would result in low likelihood scores for alleles with very low LD, since they would be a poor fit to the G6PD model. This is also an issue for alleles older than G6PD or in regions with higher rates of recombination. Since these are undesired properties, Wang et al. [4] set the likelihood equal to its maximum value for each FRC value that is between 0 and F(X)+0.1. Their test statistic is then the average log likelihood of selection, ALnLH, for each allele at the focal site given the model:

where

Here, Y_i _is the FRC at site i, X_i _is the distance from site i to the focal site, F is the expected value of FRC as a function of the distance from the focal site, N is the number of sites, and σ^2 ^is the variance of g over the entire empirical distribution.

They calculate ALnLH for each allele at each site with a homozygote minor allele frequency of greater than 0.05. From the empirical distribution, they determine the average and standard deviation of ALnLH scores. Candidates for positive selection are those SNPs where one allele has an ALnLH score of 2.6 SD above the mean while the other allele has a score of less than 1 SD above the mean. In their 2006 study, these criteria included the top 1.6% of the empirical distribution [4]. We determined the details of this algorithm from source code provided by Eric Wang (personal communication).

## Authors' contributions

CH, HH, and AR designed the study and participated in the data analysis. CH and AR developed the coalescent simulation algorithm. CH implemented the coalescent algorithm and wrote the manuscript with extensive input and feedback from coauthors. All authors read and approved the final manuscript.

## References

[B1] The International HapMap Consortium A haplotype map of the human genome Nature 2005 437 1299 1320 10.1038/nature04226 16255080 PMC1880871

[B2] Li JZ Absher DM Tang H Southwick AM Casto AM Ramachandran S Cann HM Barsh GS Feldman M Cavalli-Sforza LL Myers RM Worldwide human relationships inferred from genome-wide patterns of variation Science 2008 319 1100 1104 10.1126/science.1153717 18292342

[B3] Sabeti PC Reich DE Higgins JM Levine HZ Richter DJ Schaffner SF Gabriel SB Platko JV Patterson NJ McDonald GJ Detecting recent positive selection in the human genome from haplotype structure Nature 2002 419 832 837 10.1038/nature01140 12397357

[B4] Wang ET Kodama G Baldi P Moyzis RK Global landscape of recent inferred Darwinian selection for Homo sapiens Proc Natl Acad Sci USA 2006 103 135 140 10.1073/pnas.0509691102 16371466 PMC1317879

[B5] Voight BF Kudaravalli S Wen X Pritchard JK A map of recent positive selection in the human genome PLoS Biol 2006 4 e72 10.1371/journal.pbio.0040072 16494531 PMC1382018

[B6] Sabeti PC Varilly P Fry B Lohmueller J Hostetter E Cotsapas C Xie X Byrne EH McCarroll SA Gaudet R Schaffner SF Lander ES Frazer KA Ballinger DG Cox DR Hinds DA Stuve LL Gibbs RA Belmont JW Boudreau A Hardenpol P Leal SM Pasternak S Wheeler DA Willis TD Yu F Yang H Zeng C Gao Y Hu H Genome-wide detection and characterization of positive selection in human populations Nature 2007 449 913 918 10.1038/nature06250 17943131 PMC2687721

[B7] Pickrell JK Coop G Novembre J Kudaravalli S Li JZ Absher D Srinivasan BS Barsh GS Myers RM Feldman MW Pritchard JK Signals of recent positive selection in a worldwide sample of human populations Genome Res 2009 19 826 837 10.1101/gr.087577.108 19307593 PMC2675971

[B8] Hawks J Wang ET Cochran GM Harpending HC Moyzis RK Recent acceleration of human adaptive evolution Proc Natl Acad Sci USA 2007 104 20753 20758 10.1073/pnas.0707650104 18087044 PMC2410101

[B9] Schaffner SF Foo C Gabriel S Reich D Daly MJ Altshuler D Calibrating a coalescent simulation of human genome sequence variation Genome Res 2005 15 1576 1583 10.1101/gr.3709305 16251467 PMC1310645

[B10] Stephens M Donnelly P A comparison of bayesian methods for haplotype reconstruction from population genotype data Am J Hum Genet 2003 73 1162 1169 10.1086/379378 14574645 PMC1180495

[B11] Scheet P Stephens M A fast and flexible statistical model for large-scale population genotype data: applications to inferring missing genotypes and haplotypic phase Am J Hum Genet 2006 78 629 644 10.1086/502802 16532393 PMC1424677

[B12] Teshima KM Przeworski M Directional positive selection on an allele of arbitrary dominance Genetics 2006 172 713 718 10.1534/genetics.105.044065 16219788 PMC1456198

[B13] Przeworski M Coop G Wall JD The signature of positive selection on standing genetic variation Evolution 2005 59 2312 2323 10.1554/05-273.1 16396172

[B14] Kaplan NL Darden T Hudson RR The coalescent process in models with selection Genetics 1988 120 819 829 3066685 10.1093/genetics/120.3.819 PMC1203559

[B15] Braverman JM Hudson RR Kaplan NL Langley CH Stephan W The hitchhiking effect on the site frequency spectrum of DNA polymorphisms Genetics 1995 140 783 796 7498754 10.1093/genetics/140.2.783 PMC1206652

[B16] Griffiths RC The frequency spectrum of a mutation, and its age, in a general diffusion model Theor Popul Biol 2003 64 241 251 10.1016/S0040-5809(03)00075-3 12948685

[B17] Coop G Griffiths RC Ancestral inference on gene trees under selection Theor Popul Biol 2004 66 219 232 10.1016/j.tpb.2004.06.006 15465123

[B18] Spencer CC Coop G SelSim: a program to simulate population genetic data with natural selection and recombination Bioinformatics 2004 20 3673 3675 10.1093/bioinformatics/bth417 15271777

[B19] Slatkin M Simulating genealogies of selected alleles in a population of variable size Genet Res 2001 78 49 57 10.1017/S0016672301005183 11556137

[B20] Hudson RR Antonovics DJFaJ Gene genealogies and the coalescent process Oxford Surveys in Evolutionary Biology 1990 7 Oxford: Oxford University Press 1 44

[B21] Przeworski M The signature of positive selection at randomly chosen loci Genetics 2002 160 1179 1189 11901132 10.1093/genetics/160.3.1179 PMC1462030

[B22] Hinds DA Stuve LL Nilsen GB Halperin E Eskin E Ballinger DG Frazer KA Cox DR Whole-genome patterns of common DNA variation in three human populations Science 2005 307 1072 1079 10.1126/science.1105436 15718463

[B23] Nachman MW Crowell SL Estimate of the mutation rate per nucleotide in humans Genetics 2000 156 297 304 10978293 10.1093/genetics/156.1.297 PMC1461236

